# The Behavior of Glass Fiber Composites under Low Velocity Impacts

**DOI:** 10.3390/polym15234549

**Published:** 2023-11-27

**Authors:** Iulian Păduraru, George Ghiocel Ojoc, Horia Petrescu, Iulia Graur, Cătălin Pîrvu, Lorena Deleanu

**Affiliations:** 1 “Dunărea de Jos” University of Galati, Faculty of Engineering, Department of Mechanical Engineering, 800008 Galati, Romania; iulian.paduraru@ugal.ro; 2 National University of Science and Technology “Politehnica”, Faculty of Industrial Engineering and Robotics, 060042 Bucharest, Romania; horia.petrescu@upb.ro; 3 “Dunărea de Jos” University of Galati, Transfrontier Faculty, Department of Applied Sciences, 800008 Galati, Romania; iulia.graur@ugal.ro; 4 National Institute for Aerospace Research “Elie Carafoli”, 061126 Bucharest, Romania; pirvu.catalin@incas.ro

**Keywords:** glass fiber composite, bicomponent epoxy resin, quadriaxial fabrics, low velocity impact, hemispherical hardened steel impactor, maximum force during impact

## Abstract

This paper presents experimental results on the behavior of a class of glass fiber composites under low velocity impacts, in order to analyze their usage in designing low velocity impact-resistant components in car and marine industries. Also, a finite element model at the meso level (considering yarn as a compact, homogenous and isotropic material) was run with the help of Ansys Explicit Dynamics in order to point out the stages of the failure and the equivalent stress distribution on the main yarns in different layers of the composite. The composites were manufactured at laboratory scale via the laying-up and pressing method, using a quadriaxial glass fiber fabric (0°/+45°/90°/−45°) supplied by Castro Composites (Pontevedra, Spain) and an epoxy resin. The resin was a two-component resin (Biresin^®^ CR82 and hardener CH80-2) supplied by Sika Group (Bludenz, Austria). The mass ratio for the fabric and panel was kept in the range of 0.70–0.77. The variables for this research were as follows: the number of layers of glass fiber fabric, the impact velocity (2–4 m/s, corresponding to an impact energy of 11–45 J, respectively) and the diameter of the hemispherical impactor (Φ10 mm and Φ20 mm) made of hardened steel. The tests were performed on an Instron CEAST 9340 test machine, and at least three tests with close results are presented. We investigated the influence of the test parameters on the maximum force (F_max_) measured during impact, the time to F_max_ and the duration of impact, t_f_, all considered when the force is falling to zero again. Scanning electron microscopy and photography were used for discussing the failure processes at the fiber (micro) and panel (macro) level. At a velocity impact of 2 m/s (corresponding to an impact energy of 11 J), even the thinner panels (with two layers of quadriaxial glass fiber fabric, 1.64 mm thickness and a surface density of 3.51 kg/m^2^) had only partial penetration (damages on the panel face, without damage on panel back), but at a velocity impact of 4 m/s (corresponding to an impact energy of 45 J), only composite panels with six layers of quadriaxial fabric (5.25 mm thickness and a surface density of 9.89 kg/m^2^) presented back faces with only micro-exfoliated spots of the matrix for tests with both impactors. These results encourage the continuation of research on actual components for car and naval industries subjected to low velocity impacts.

## 1. Introduction

By increasing their performance (increasing load, higher velocity and higher temperature), technical systems have also increased the risk of impact with machined parts or components of other systems. Thus, the interest in impact protection has increased due to the reasonable allocation of financial resources to research and develop impact protection technologies and components in the context of the observed failures of some components under impact.

This type of impact of low velocity can have significant effects on vehicle components and structure. Low impact velocities generate concentrated forces at certain vulnerable points. It is therefore important to understand how different materials behave under impact forces in order to develop appropriate protective solutions. Low velocity impact, with values between 1 m/s and 10 m/s, is not only specific to processes associated with automotive operation but may also be encountered in a variety of other fields: in food, chemical or pharmaceutical industries, in handling, transport or packaging of products and materials, and in the aircraft and naval industry.

Composites reinforced with glass fibers in an epoxy resin as the matrix have been studied in terms of their response to low velocity impact since the 1970s [1]. Khan et al. conducted impact tests on glass fiber/vinyl ester resin and glass fiber/epoxy resin reinforced pipes, finding that the energy required to initiate damage was higher for pipes based on epoxy resin compared to pipes made of vinyl ester resin. These findings explain that low velocity impacts can induce significant failure in the material, causing serious degradation of the otherwise excellent mechanical properties of the composites and premature and unexpected failure of the entire system [2].

Giansin et al. [3] tested a glass fiber epoxy composite for the aerospace industry at low velocity impact using small samples of 70 mm × 70 mm. Laminates have four different yarn orientations, with 32 sublayers formed by 8-layer groups of 0°/90°/+45°/−45°, 8 layers of 0°/90°/90°/0°, 16 layers of +45°/−45°, and 32 layers of unidirectional fabric. The impact energy was 75–225 J, using a hemispherical striker of 20 mm in diameter. The experimental results showed that plates with unidirectional fiber orientation failed due to shear stresses, while no penetration occurred for the panel made of 0°/90°/90°/0° fabric and +45°/−45° fabric due to a better energy transfer for these yarn architectures.

The effect of increasing impact energy on composites made of basket-woven glass fiber fabrics and epoxy resin, using a laboratory technology with vacuum-assisted resin transfer molding, was reported in [4], with the resulting thickness of tested plates being 4.57–5.33 mm. The tests were performed on an Instron Dynatup impact drop tower machine, with impact velocities of 1.30–3.42 m/s and an impact energy range of 0.72–7.36 J. Their finite element model gave results in agreement with the experiment: the impactor considered was made of hardened steel and the meshing network was adapted to solve a friction contact between the impactor and the composite (a value of 0.3, constant, was adopted for the friction coefficient).

Bilisik et al. [5] pointed out the introduction of woven, braided, knitted and non-woven 2D fabrics in composites, but fatigue and impact produce delamination as one of the main failures, along with fiber breakage, underlining the importance of the matrix quality. In impact processes, delamination is a method of energy absorption and sometimes helps to reduce the breakage of the reinforcement [6].

Wang Y. et al. [7] presented a finite element analysis to investigate the impact on multi-ply fabric panels with different orientations. Influencing factors, such as impact velocity, number of plies and impactor dimension, were taken into account. The yarn orientation affected the energy-absorbing capacity of the multi-ply fabric panels. The authors estimated that diagonal yarn architecture increased the energy-absorbing capacity, as compared to yarn parallel to the sides of the panels. The model pointed out that the stacking sequence of oriented layers influences the absorbed energy.

This research presents a simulation of the impact, with the help of the finite element method, impact also produced experimentally on quadriaxial glass fiber panels with different thicknesses (or number of layers). This paper also presents the test results and a discussion on the failure mechanisms, as noticed by the authors with the help of high-resolution photos and scanning electron microscopy, pointing out the influence of impact velocity, number of fabric layers and the dimension of the hemispherical impactor. The objective of this study is to formulate recommendations for the manufactured panels in terms of partial or total penetration for the tested ranges of parameters: 2–4 m/s for the impact velocity, 11–44 J for impact energy and 1.64–5.25 mm thickness of the panels.

## 2. Materials and Methods

Analysis of technological solutions for multi-axial glass fiber woven and unidirectional composites at low velocity impact has been investigated in several studies [8,9,10,11,12]. Non-woven fabric is a type of fabric that is not woven or knitted in the traditional way. It is produced by bonding or interlocking fibers, long or short, together using mechanical (stitching, etc.), chemical or heat-reacting processes. From a mechanical point of view, in woven and knitted fabrics, yarns consume some of their resistance for curling or crimping. Unidirectional fabrics preserve this strength for facing external load, but the technology and auxiliary materials for keeping yarns together have a great importance.

**Figure 1:**
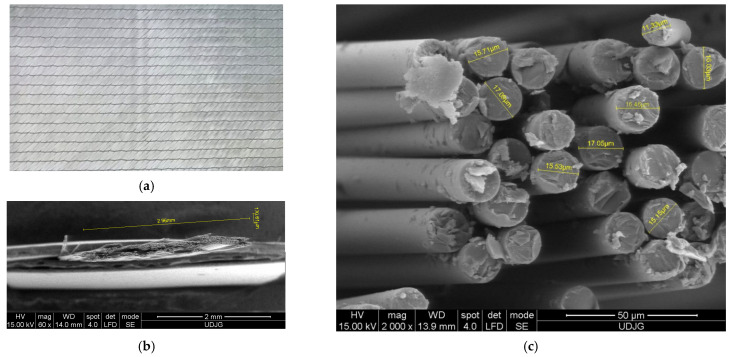
Aspects of the glass fiber fabric: (**a**) front view of the quadriaxial fabric, (**b**) dimensions of the yarn, (**c**) measurements of the glass fibre diameters (fibres in the same yarn, cut with scissors).

Based on research reported in [7,13,14,15,16,17,18], the authors selected a glass fiber fabric or a prepreg with four sublayers, with yarn orientation 0°/+45°/90°/−45°. This fabric has the trade name “1200 g/m^2^ Quatriaxial Glass Cloth (0°/+45°/90°/−45°) 127” and is made of E-glass fibers. The product was purchased from Castro Composites (Pontevedra, Spain) [19]. Figure 1 presents aspects of the fabric: a) macro aspects of the fabrics, b) detail with the dimensions of the yarn and c) a micro-view of fibers in a yarn, with cross-section diameter measurements. Table 1 presents the average composition of glass fibers for the cross-section and for the fiber jacket, and it may be noticed that Boron (B), Carbon (C), Aluminum (Al), Silicon (Si) and Calcium (Ca) predominate, with traces of Iron (Fe), Zinc (Zn) and Titanium (Ti), a composition that could be positioned between E-glass and S-glass, its particularity being assigned to the extraction area of the raw material.

After a review of the available literature in the field of glass fiber composite resins, the authors selected Biresin^®^ CR82 two-component resin in combination with CH80-2 hardener (production year: 2021). The resin and hardener were purchased from Sika Group [21]. Biresin^®^ CR82 resin is an epoxy resin recognized for its excellent adhesion to glass fibers. This resin offers high resistance to mechanical load and a high durability, making it ideal for use in applications with high strength and durability requirements [22]. CH80-2 hardener is a curing agent compatible with Biresin^®^ CR82 resin, which imparts chemical and mechanical properties to the finished composite. This hardener improves impact strength and adhesion between the resin and fibers, ensuring a uniform distribution of the hardener in the composite and a proper curing reaction. The selection of this resin is based on the advantages it offers in terms of mechanical properties, impact strength and adhesion, all crucial factors for obtaining high-quality composites. Table 2 presents the characteristics of the resin components for preparing the mixture of the layed-up composites. Table 3 presents mechanical and thermal properties of the epoxy resin, obtained with different hardeners. The selected hardener, Biresin^®^ CH80-2, is recommended by the producer as having a better impact resistance, a characteristic that authors were looking for to produce the panels.

The laboratory-scale technology is able to produce composite panels using the laying-up method, pressing, a natural ageing duration and a thermal treatment. After the thermal treatment, the panels can be tested. The resulting panels have their characteristics (thickness and mass ratio fabric/panel) in a narrow range (Table 4).

**Figure 2:**
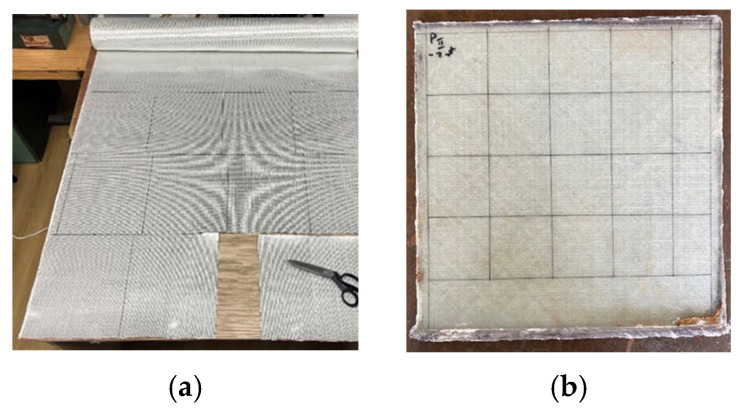
Images during the laboratory-scale production of the samples: (**a**) cutting the sheets of 300 mm × 300 mm, (**b**) a panel marked for cutting 60 mm × 60 mm samples.

The operations for producing the panels are as follows: cutting sheets of fabrics of 300 mm × 300 mm, weighing each package of sheets (2 sheets, 4 sheets and 6 sheets in a panel), waxing the active zone of the press for easier removal of the obtained panel, mixing the resin components in the recommended ratio, laying-up each sheet with the resin, pressing and maintaining this for at least 8 h, extracting the sheets from the press mold, verifying and polishing the edges, natural ageing for 7 days, a heat treatment for structure stabilization (6 h at 60 °C in an oven with controlled temperature), quality control and measurements (thickness in four points, mass of the panel and calculation of the mass ratio between the fabrics and the obtained panel), and tailoring and cutting with high-speed disks in the dry regime of samples of 60 mm × 60 mm from each larger panel (Figure 2) and their codification. For an easier extraction of the panel from the press, a wax type CIREX CP 10 was used (the supplier being RomPolimer Composites, Romania). The production time is not significantly influenced by the number of fabric layers in the composites.

**Figure 3:**
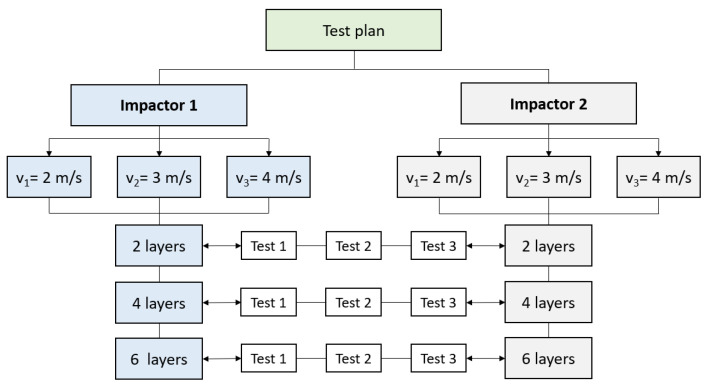
The test plan of the research. Both impactors are made of hardened steel with a hemispherical head; impactor 1 is 10 mm in diameter and impactor 2 is 20 mm in diameter.

Impact tests were conducted at room temperature using the Instron CEAST 9340 drop tower impact system [23]. Three samples under the same impact test conditions were used. The test plan is presented in Figure 3. This plan will help to establish the influence of impact velocity, hemispherical impactor size and the number of quadriaxial layers in a composite panel.

Two different diameters of hemispherical hardened steel impactor were used; impactor 1 is 10 mm in diameter, while impactor 2 is 20 mm in diameter. The impactor velocity varies between 2 m/s and 4 m/s, with three different values noted as v_1_ = 2 m/s, v_2_ = 3 m/s and v_3_ = 4 m/s. The nominal impact energies corresponding to the above-mentioned velocities are 11 J, 23 J and 44 J, respectively.

**Figure 4:**
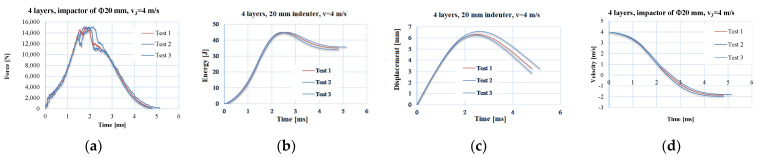
Test repeatability on 4-layer panels with the same impactor and impact velocity. An example of a test repeated three times under the same conditions (impactor of Φ20 mm, 4-layer panel and v_3_ = 4 m/s): (**a**) force—time graphics, (**b**) energy—time graphics, (**c**) displacement—time graphics, (**d**) velocity—time graphics.

The test machine is equipped with an anti-rebound system, a temperature-controlled working enclosure and a height adjustment system from which the impactor starts falling in order to vary its kinetic impact energy. The characteristics of the machine are as follows: energy range of 0.30–405 J, impact velocity of 0.77–4.65 m/s, drop height of 0.03 to 1.10 m and impactor mass between 1 kg and 37.5 kg. Figure 4 presents the curves of the discussed parameters in the following section as a function of time—(a) force, (b) energy, (c) displacement and (d) velocity—in order to point out the repeatability of the tests and also to reflect the composite quality.

## 3. Finite Element Model and Results of the Simulation

Simulations were run to highlight the possibility of modeling the impact at the meso level, taking into account the behavior of unidirectional yarns with isotropic characteristics, an acceptable simplification because the architecture of yarns in multi-axially oriented substrates (0°/45°/90°/−45°) gives some uniformity to the mechanical characteristics, at least in the plane of the fabric; thus, on the basis of validation criteria, the model can be used to evaluate the low velocity impact resistance for certain ranges of parameters, such as the panel area, number of layers and impact velocity, in the vicinity of parameters already used on the tested panels.

**Figure 5:**
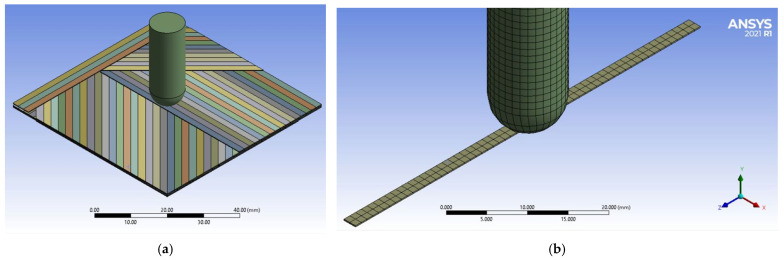
Details of the model: (**a**) yarn architecture (orientation of several yarns on each sublayer); (**b**) mesh network of the impactor and a yarn.

The simulation is performed for a panel having the same dimensions as the actually tested samples, 60 mm × 60 mm, with two layers of quadriaxial layers (meaning 8 sublayers of single yarn arrangement). The geometry of the model is given in Figure 5.

The glass fibers are known as having an anisotropic feature, but in this model, the yarn is considered a monobloc body, with isotropic and homogenous character. Actually, due to the architecture of the yarns, for the four sublayers in the fabric, at orientations of 0°, +45°, 90° and −45°, there exists a quasi-isotropic character of the panel, generated by the yarns’ different orientations.

In order to minimize or to avoid hour glassing in finite element analyses, specific techniques and algorithms are used, such as re-meshing methods, mesh adaptation or the erosion of elements with excessive deformations [24,25]. In numerical analyses, the use of uniform discretization networks is preferred as it brings benefits in terms of efficiency, accuracy and solution stabilization [26].

This simulation allows for the detection (“visualization”) of failure mechanisms that can only be observed on real specimens as a result at the end of a test [27].

In order to simulate the system behavior as realistically as possible, the material models include two aspects:

- the failure criterion, which describes the breaking, detachment and cracking when the critical value of a material characteristic (for instance, for stress or strain) is exceeded;
- the stress/strain evolution model for each material used for the solid bodies, which could include a particular behavior (elastic, plastic, viscous or combined) of the materials.

Validation criteria could be as follows:

- quantitative: here, the number of broken layers, size of the indentation or orifice for total penetration, impactor displacement over time and size of the delamination (or detachment of layers and sublayers), observable on the simulation by virtually sectioning the model,
- qualitative: including the geometric shape of the indentation or orifice produced by the impactor, and the shape of the delamination.

Simulations are useful for the identification of impact stages, which cannot be noticed by post-mortem studying the target damage due to the very small time intervals over which it occurs, and for analysis of the state of stresses and strains for different targets as a function of the thickness or number of layers, impact velocity and energy.

The paper [28] examines the effect of yarn cross-sectional shape to explain the resin flow through gaps between yarns in composites made of woven fabrics. The findings may be used to optimize the design of such composite materials by selecting the appropriate yarn cross-section shape. The study also highlights the importance of accurate modeling of yarn cross-sections in the numerical prediction of textile permeabilities. Cross-sections of a yarn could be considered circular, with a flat central body and narrowed edges, elliptical, with a flat central body and rounded sides, biconvex, similar to a lens, or rectangular [13]. For this model, the yarns are considered to have a rectangular cross-section, with dimensions equal to that of a rectangle that include the actual shape of the yarn. The model consists of 265 bodies, of which 264 are yarns and 1 is the hemispherical impactor:

- sublayers 0° and 90° have 30 yarns on each layer;
- sublayers +45° and −45° have 36 yarns on each layer.

For the model with the 10 mm impactor, the number of nodes is 107,017 and the number of elements is 40,496, and there are 117,195 nodes and 49,896 elements for the model with the 20 mm impactor. Each yarn in a sublayer is fixed on its lateral edge so that this end section does not move. The pattern of the panel is structured as follows: 8 unidirectional yarn sublayers, with orientation 0°, +45°, 90° and −45°, and again with 0°, +45°, 90° and −45°, successively. The yarns are 0.2 mm in thickness and 2 mm in width.

In order to reduce the space in which the simulation takes place, the impactor has a shorter length of its cylindrical part. Its density was adopted to reach 2.99 kg for the Φ10 mm impactor and 3.7 kg for the impactor with Φ20 mm, as is the case of actual impactors used in testing the panels.

**Figure 6:**
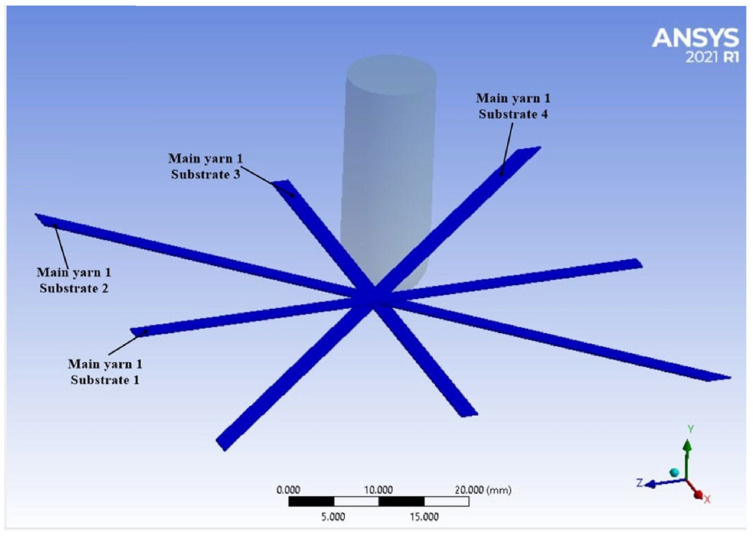
The codification of the yarns for a sequence of 4 sublayers (yarns are in their initial position, without being stressed).

The codes of the analyzed main yarns are preserved throughout this analysis, as in Figure 6. Main yarn is the first one near the plane of symmetry of the model (through the impactor axis and parallel to one panel side) and it will bear the direct contact of the impactor. In the following figures, the number of the substrate on which the yarn is located is added.

Sublayers are connected as “bonded”, and the yarns on the same sublayer are also “bonded” but have a detaching condition, namely, “breakable” (with “stress criteria”) when imposed conditions are fulfilled: here, when the normal and shear values are in agreement with the following relationship:

(σ_n_/σ_n_limit_)^n^ + (|σ_s_|/σ_s_limit_)^m^ ≥ 1, (1)

where σ_n_ is the normal stress in the analyzed connection, σ_s_ is the shear stress in the same point, σ_n_limit_ is the tensile strength at breakage, σ_s_limit_ is the shear strength at breakage and here, the exponentials are considered n = m = 2 [29,30]. For this model, the values involved in Relationship (1) are σ_n_limit_ = 90 MPa and σ_s_limit_ = 60 MPa. These values are characteristics for a high-quality epoxy resin used in composites.

**Figure 7:**
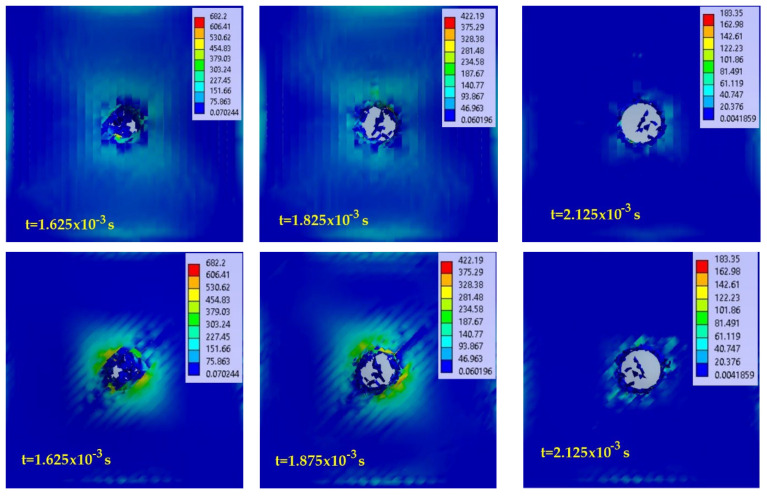
Virtual images from simulation with equivalent stress distribution (in MPa), at different moments, for the panel made of 2 layers of quadriaxial fabric (meaning 8 sublayers) hit by the impactor with Φ10 mm at the impact velocity of v_3_ = 4 m/s (up—panel face, down—panel back).

**Figure 8:**
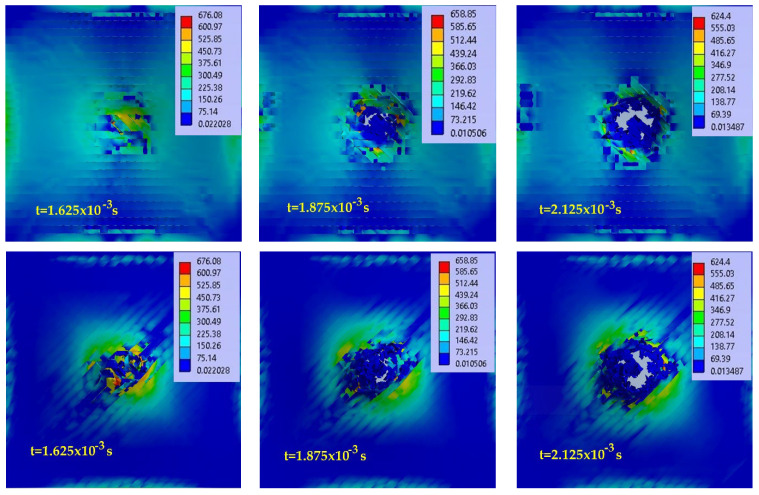
Images from simulation with equivalent stress distribution (in MPa), at different moments, for the panel with 2 layers (8 sublayers) with the impactor of Φ20 mm at impact velocity v_3_ = 4 m/s (up—panel face, down—panel back).

Figure 7 presents virtual images from the simulation for different time moments, including the equivalent stress distributions for the 2-layer panel, impacted with the Φ10 mm impactor: up—the panel face, down—the panel back. Figure 8 presents the same panel but hit with the Φ20 mm impactor. Each image has its own color scale for the equivalent stress, given in MPa.

For the total penetration characterizing the 2-layer panel impacted with a hemispherical impactor, the impact stages noticed during simulation are as follows:

- the stressing of sublayers without breaking yarns is a very short stage, lasting 10^−4^ to 10^−3^ s, longer than in a ballistic impact, which is of the order of 10^−5^–10^−6^ s [20], but the equivalent stress values reach high values, close to the breaking limit, and the fiber strain approaches the failure criterion value, which is, here, the equivalent plastic deformation at break;
- the initiation of yarn breakage and delamination between substrates from the moment when the value set for the yarn failure criterion is reached (here equivalent plastic strain at break—EPS); higher delamination is observed between the last substrates due to their higher strains;
- the stage with successive breakage of all yarns (the breakage of one or more yarns can be achieved in the simulation between the selected moments, and therefore, the equivalent stress graphs on the yarns can have values lower than the breakage limit);
- the stage with equivalent stresses on the main yarns being lower than the stress limit of the yarns but with values that can detach adjacent yarns in the same sublayer and yarns situated on adjacent sublayers; this is because the stress exceeded the failure criterion for the composite matrix given in Relationship (1), simulated by the detachment of nodes at certain values of tensile or shear stress between yarns (in this study, the matrix has zero thickness, but it manifests its mechanical properties);
- the stage with equivalent stress below the values that cause the matrix to fail; no more yarn detachment occurs and the impact process can be considered complete.

**Figure 9:**
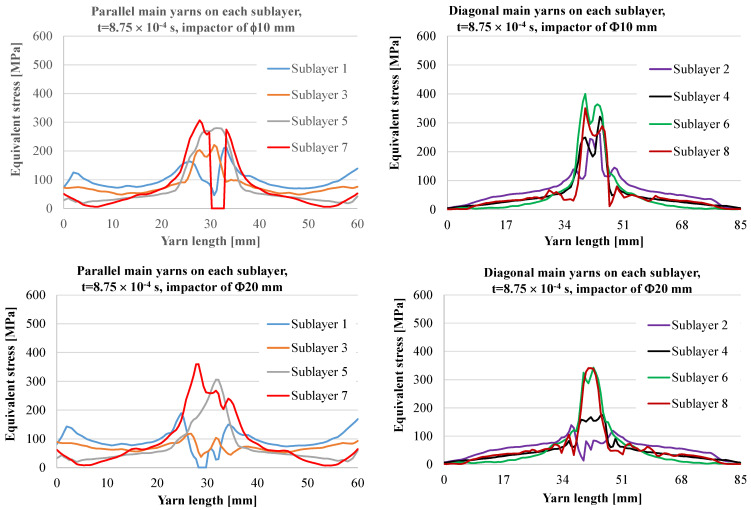
Equivalent stress distribution for time moment *t* = 8.75 × 10^−4^ s.

**Figure 10:**
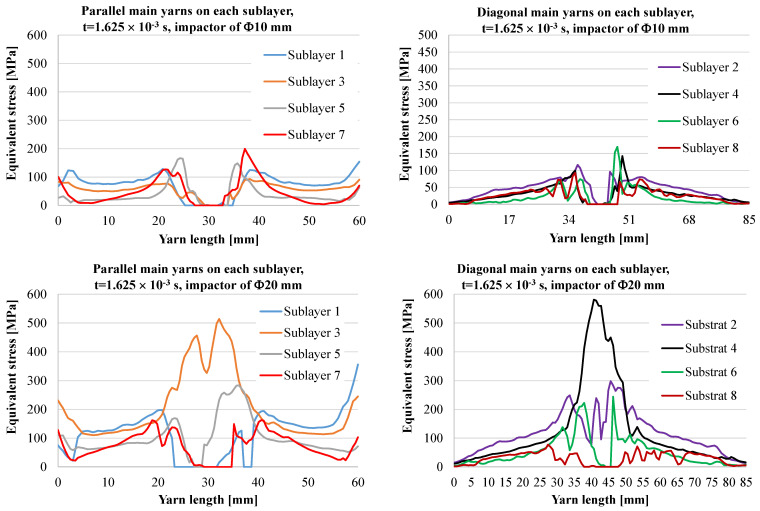
Equivalent stress distribution for moment *t* = 1.625 × 10^−3^ s.

Figure 9 and Figure 10 present graphs of the equivalent stress on a main yarn on each of the 8 sublayers of the panel for two moments during the simulation.

From the 20 time steps of the simulation, the authors selected this one, t = 8.75 × 10^−4^ s, as it points out the differences in the behavior of the panel and differences caused by the impactor dimension, the only parameter which changed in these two simulated cases. In Figure 9, graphs in the upper line are for the impactor of the 10 mm diameter and those in the bottom line are for the impactor of 20 mm. As the analyzed main yarns have different lengths (the yarns parallel to panel side—60 mm, the same as the actual ones, and the diagonal yarns—85 mm length), each type of yarn is given in separate graphs, but the axes of the impact is at half-length for each one, either parallel or diagonal yarns. At this moment, for the panel hit by the Φ10 mm impactor, the main yarn on sublayer 7 is already broken and the segment between zero stress suggests a yarn break in two points not symmetrically positioned. This fragment will be pushed by the impactor head. The diagonal yarns are not yet broken, but high values of the equivalent stress are noticed. Delamination is possible between sublayer 1 and 2 on a larger area and even towards the fixed end of the yarns and near the contact. Diagonal yarns have lower values of stress along them because they are longer.

For the same moment, the impactor of Φ20 mm diameter produces different distributions of equivalent stress. The main yarn on sublayer 1 is already broken and also sublayer 7 has high values towards the yarn limit. Diagonal main yarns present high values for equivalent stress for the sublayers 6 and 8.

The same notations are preserved for Figure 10, which presents equivalent stress distributions at the moment *t* = 1.625 × 10^−3^ s. For the impactor of 10 mm in diameter, all main yarns are broken and delamination is visible near the contact due to the bending of the yarns when the impactor pushes the panel and/or advances through the panel. The impactor of 20 mm in diameter does not break all of the yarns; those on sublayers 2, 3 and 4 are still intact but highly stressed. The yarns on the last layers are prone to be broken as they have no solid support on their back.

## 4. Experimental Results

The following parameters, obtained from the experimental campaign, are discussed here:

- the maximum force, F_max_,
- the duration till F_max_, t_(Fmax)_,
- the duration of the impact, considered from the last recorded values F = 0, from which the force increases, marking the moment, t_0_, till the moment when force, after its evolution through F_max_, is again 0; this happens at moment t_f_.

**Figure 11:**
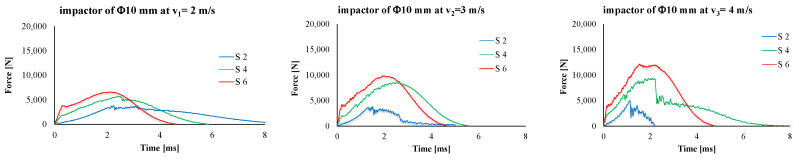

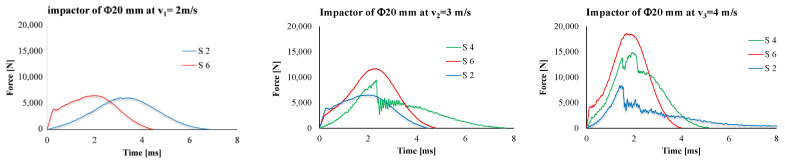
Typical force–time curves for the test conditions (up—impactor of 10 mm in diameter, down—impactor of 20 mm in diameter; impact velocity increases from left to right).

Figure 11 presents typical force–time curves. Total penetrations are characterized by a saw-teeth aspect (see, in particular, the panels made of two layers of quadriaxial fabric, hit by the smaller diameter impactor). Partial penetrations are characterized by an asymmetrical bell shape. The maximum value of the force increases with the number of layers of the hit panels, and the same dependence on velocity is evident. For the same impactor, the maximum values for F_max_ were obtained for the highest velocity (4 m/s): 12,000 N for the Φ10 mm impactor and 18,000 N for the impactor of Φ20 mm.

**Figure 12:**
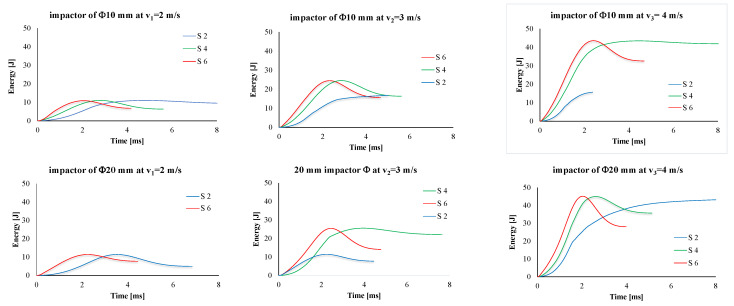
Influence of number of layers of quadriaxial fabrics and of impact velocity on the energy–time curve (up—impactor of Φ10 mm, down—impactor of Φ20 mm).

Typical absorbed energy–time curves are given in Figure 12. The slope of the energy is greater for thicker panels; thus, these are capable of absorbing the impact energy faster.

**Figure 13:**
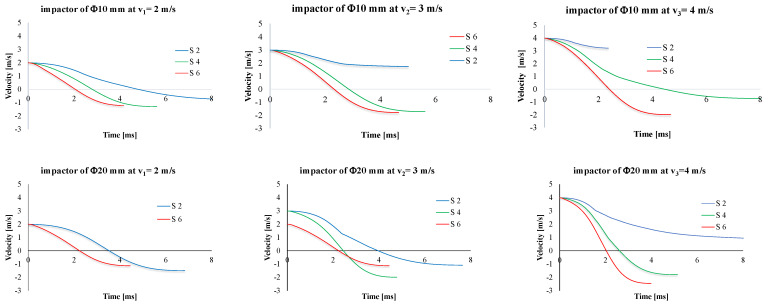
Influence of number of layers of quadriaxial fabric and impact velocity on the velocity–time curve (up—impactor of Φ10 mm, down—impactor of Φ20 mm).

Figure 13 presents the typical curves for the velocity of the impactor (up—for the impactor of Φ10 mm, down—for the impactor of Φ20 mm). The slopes of the plots are greater for thicker panels. A null value of the velocity means that the impactor is stopped and then it rebounds (the velocity becomes negative in the machine system of measurement). Total penetration is characterized by graphs that do not reach the null value during the impact, meaning the impactor is continuing its trajectory after the total penetration of the panel till the recovery (stopping) system of the machine is activated. The moment when the impactor velocity is null, t_(v=0)_, indicates the moment when the entire kinetic energy of the impactor is transferred to the composite panel. The velocity decreases and reaches zero when the impactor is stopped; then, it is rebounded and the velocity is recorded as negative (v < 0, Figure 13). When the penetration is total (meaning all of the layers are damaged and the impactor continues its trajectory with a residual velocity), the velocity–time curve has no intersection with the time axis.

**Figure 14:**
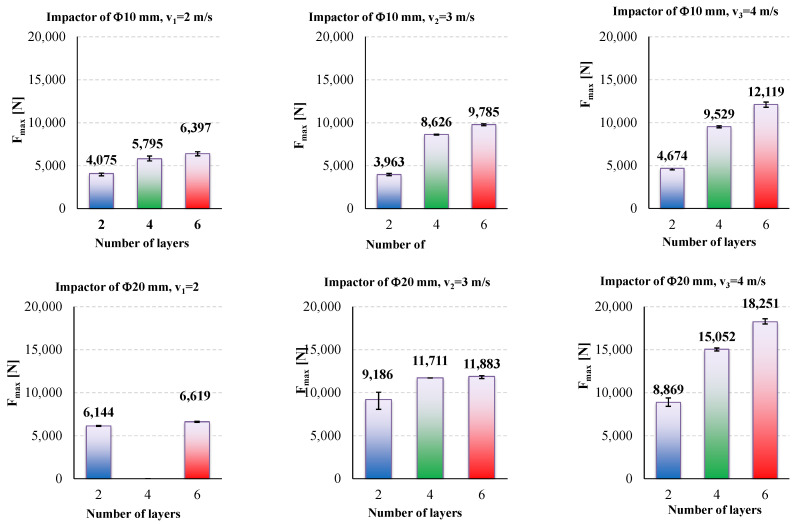
Average values and spread intervals for measured maximum force, F_max_: up—results obtained with the impactor of Φ10 mm, down—results for the impactor of Φ20 mm; the impact velocity increases from left to right.

**Figure 15:**
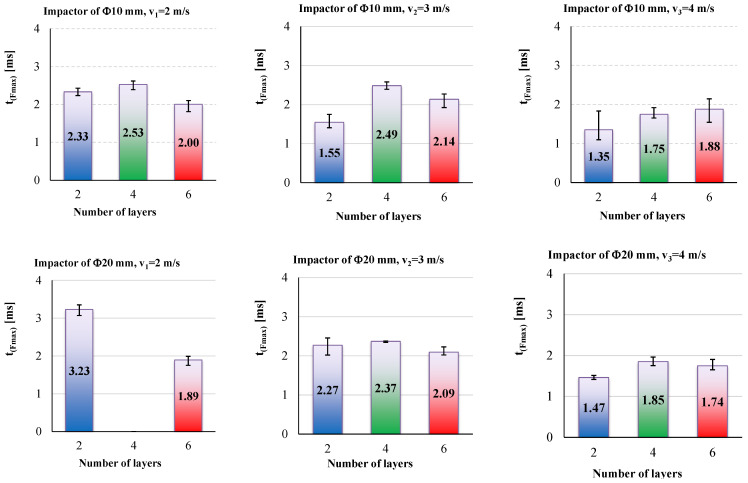
Average values and spread intervals for measured time till the maximum force, t_(Fmax)_: up—results obtained with the impactor of Φ10 mm, down—results for impactor of Φ20 mm; the impact velocity increases from left to right.

A better representation of the analyzed parameters is given in Figure 14 and Figure 15; the written value is the average, calculated with the three values recorded in the tests with the same test conditions, while the black segment represents the spread range of the same parameter, F_max_ or t_(Fmax)_. A clear increase in the impact velocity is presented by F_max_ (Figure 14), but this increase is not similar for each velocity and dimension of hemispherical impactor.

The duration of the impact till F_max_ has no clear tendency, but the values are very small, around 2 ms.

Thus, the value of maximum force, F_max_, could be used to evaluate the impact process, but the time till this value of the measured force is not relevant (at least for the tests presented here) to be in a clear dependency with the velocity and number of quadriaxial layers in the panel. But if one compares the values for the lowest velocity (v_1_ = 2 m/s) to those for the highest value (v_3_ = 4 m/s), this time till F_max_, t_(Fmax)_, is smaller for the highest impact velocity.

Table 5 and Table 6 present the average values of the discussed parameters, first for the impactor with a Φ10 mm and second for the impactor with a Φ20 mm. From these two tables, it is easy to observe the following:

- F_max_ increases with the increase in the number of quadriaxial layers: for the two-layer panels, F_max_ increases by 14.6% for v_3_ = 4 m/s as compared to the value for v_1_ = 2 m/s; for the same values of the impact velocities, a larger difference is obtained for the six-layer panel, as F_max(v3=4 m/s)_ is greater by 89.4% than F_max(v1=2 m/s)_;
- the moments when the force reaches its maximum values are larger for the lower impact velocity and shorter for higher velocities, but the difference is small, about 0.3–1.0 ms;
- t_f_ has a clear tendency to be reduced both with the increase in panel thickness and impact velocity;
- t_(v=0)_ represents the moment when the entire kinetic energy is transferred to the panel; always t_(v=0)_ < t_f_ as during the rebounding of the impactor, the impactor has to face the force produced by friction and supplementary delamination could occur by gripping the perforated layers during the rebound;
- when total perforation occurs (as for the two-layer panels impacted with v_2_ = 3 m/s and v_3_ = 4 m/s), E_max_ is lower than the nominal energy of the impact, E_N_, (here, E_N_ has the values 11 J, 25 J and 44 J, respectively).

Based on Figure 15 and the force–time and velocity–time curves, Table 7 presents the failure of the panels, grouped into three categories: PT—total penetration (the panel is not recommended for actual applications), PP—partial penetration (the panel could be recommended, of course, with a broader test plan for the number of samples and with impactor shape and dimensions close to the actual ones, PP ^⁕^—partial penetration with severe damages on the panel back, also not recommended for practical applications.

**Figure 16:**
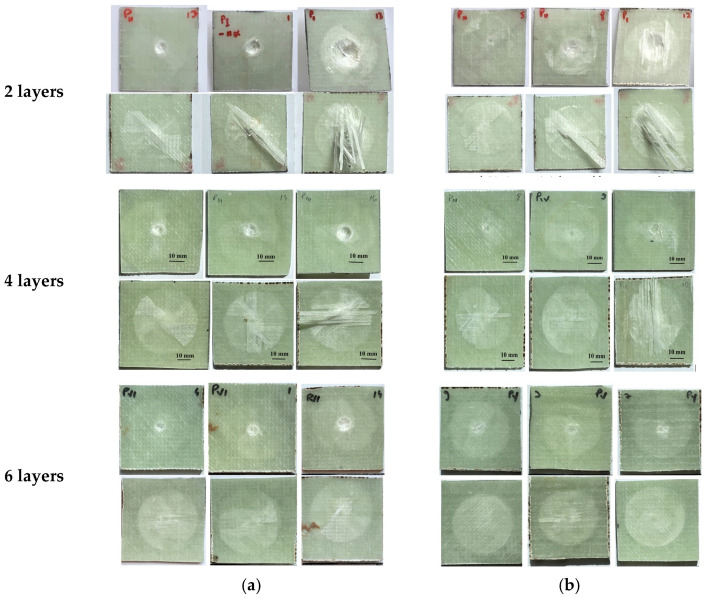
Macro views of composite panels after being tested—face (up) and back (down)—for impact velocities of v_1_ = 2 m/s, v_2_ = 3 m/s and v_3_ = 4 m/s (from left to right). (**a**) Impactor of Φ10 mm, (**b**) impactor of Φ20 mm.

Figure 16 presents in images, and very suggestively, the results of the test plan. It is obvious that panels with two layers of quadriaxial fabrics do not resist to impact caused by both impactors. For the panels made of four layers and six layers of quadriaxial fabric, the diameter of the imprint caused by the impactor of 10 mm is proportional to the impact velocity. But the impactor of 20 mm generates similar dimensions of the imprint and no dependence on impact velocity could be formulated. Considering the color change as the effect of delamination, this failure mechanism is less visible on the front of the two-layer panels hit by the impactor of 10 mm. For the six-layer panels, the delamination on the panel back seems to be similar for both impactors and unsensitive to impact velocity.

**Figure 17:**
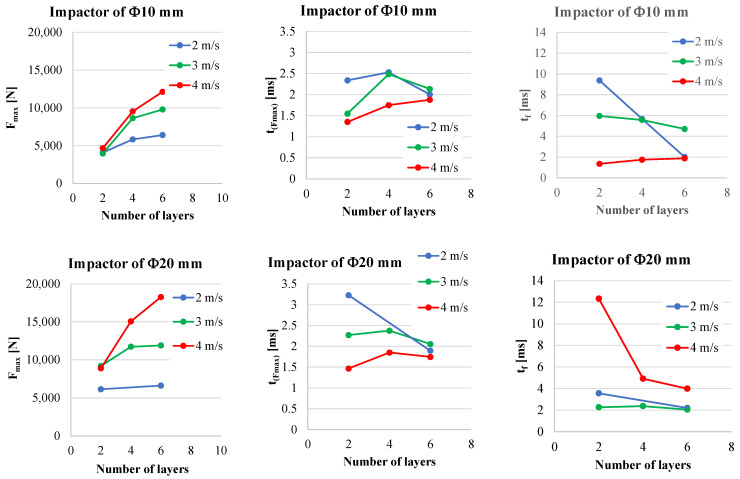
Synthetic representation of analyzed parameters of the impact, F_max_, t_(Fmax)_ and t_f_; first line is for the tests conducted with the impactor of Φ10 mm and the second line is for the tests conducted with the impactor of Φ20 mm.

Figure 17 is a synthesis of the test campaign results. The average values of the three parameters are given for both impactors. F_max_ increases with the increase in the number of quadriaxial layers but also with the increase in impact velocity. Slopes are greater for the highest velocity, v_3_ = 4 m/s. The duration till F_max_ seems to be less dependent on these parameters (number of quadriaxial layers and impact velocity). It is interesting to mention that, for both impactors, these values have the tendency to be grouped around 2 ms. The impact duration, t_f_, has no obvious dependence on impact velocity and number of quadriaxial layers that could be pointed out for the smaller impactor (with the diameter of 10 mm). For the impactor with a diameter of 20 mm, the impact duration is obviously decreasing with the number of layers only for velocity v_3_ = 4 m/s. For the smaller velocities (v_1_ = 2 m/s and v_2_ = 3 m/s), this dependence is less visible.

## 5. Failure Mechanisms

The failures that occur as a result of impact depend on many parameters, such as the shape of the impactor, impact velocity and target characteristics, e.g., fiber material(s), matrix, architecture of the layers (here, fiber orientation), etc. [31,32]. Failures include fiber breakage, matrix cracking, delamination and peeling. These different failure modes (including macro-scale composite fragmentation, deformation and cracking) could occur simultaneously under impact loading or in a very short time interval [33]. During impact, failures are initiated in composites and extend around the impact zone [34], reducing their stiffness and strength. Part of the kinetic energy of the impactor is consumed in the elastic then plastic deformation of the target, another part for overcoming friction, and finally for total or partial perforation, their share being influenced by the mechanical characteristics of the components of the composite panel.

**Figure 18:**
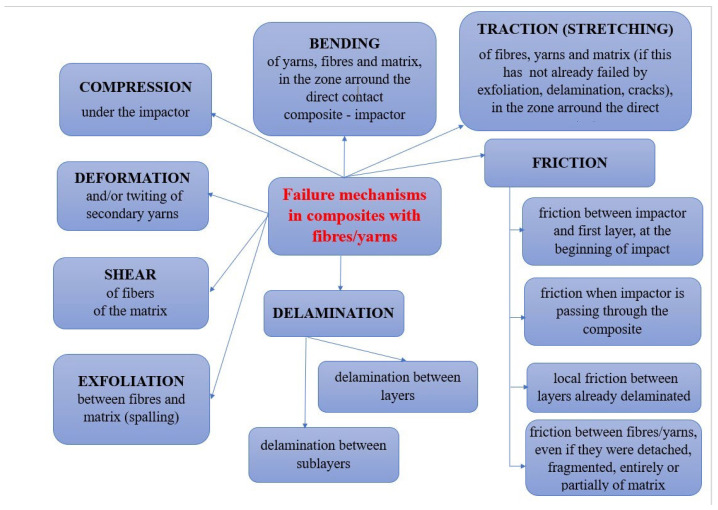
Failure mechanisms in composites with yarns and fibers, under low velocity impact, adapted from the literature [35,36,37,38,39].

This analysis provides a detailed insight into how the composite reacts under the action of impact with different variable parameters (impact velocity, impactor size and the number of fabric layers) and can provide important information about the energy absorption and failure of the respective panel (Figure 18). To underline the importance of this analysis, the recently published paper by Lopresto V. and co-workers [35] presented an overview of processes occurring in composites for the aviation and marine industry at low impact velocities and high temperatures performed with non-destructive investigative techniques. It is underlined that these materials, long fiber composites in a polymeric matrix, have a certain degree of inhomogeneity and anisotropy. Other relevant studies in failure analysis of long fiber composites are given in [36,37,38,39].

The macrophotography focuses on features visible to the naked eye, such as the texture, shape and size of visible deformations and cracks, which occur in low velocity impact-tested plates. It helps to reveal the presence of the delaminated zone(s) (via color change on the front or back of tested panels) but does not reveal microscopic details specific to the failed, micro-sized components (glass fibers, in this case).

All SEM images were obtained after the samples were gold-coated, with the help of a scanning electron microscope FEI Quanta 200 from ”Dunarea de Jos” University.

**Figure 19:**
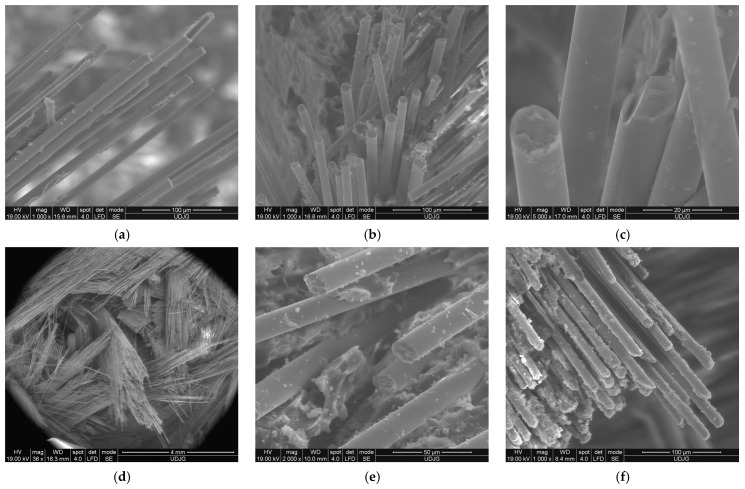
SEM images of the composite with 2 layers of quadriaxial fabric—panel face (**up**) and panel back (**down**)—after being hit with the impactor of Φ10 mm at an impact velocity v_3_ = 4 m/s.

Figure 19 shows different failure mechanisms that can occur in these glass fiber composites noticed on the SEM images of the two-layer quadriaxial fabric panels subjected to an impact velocity of v_3_ = 4 m/s, with a Φ10 mm impactor:

(a) fibres broken by shearing at different lengths: the matrix was already detached from the fibers as its mechanical characteristics are much lower; breakage occurred at different positions on the fibers (not like a straight cut resulting from a knife impact) by shear, which is more visible in image b); the fibers are stressed above the limit of the matrix, and thus, the matrix was already detached from the fibers, weakening the zone; the image is from the face of layer 1;
(b) an image of broken fibers near the edge of the imprint; two different orientations of fibers are visible, with sections of the shear breakage being almost perpendicular to the fiber length;
(c) detail with shear-cut fibers, one having a chippy edge;
(d) a low magnification (×30) that points out the different orientations of the yarns in the quadriaxial architecture; the impactor has penetrated the entire panel and broken the yarns and fibers that subsequently are bent and forced by the impactor trajectory through the orifice;
(e) detail with shear-cut fibers, with small spots of the matrix still bonded to the fibers; bellow the ”naked” fibers, the fragmented matrix is visible;
(f) the cut fibers in a yarn at different lengths suggest a statistical process of being broken; it is visible that the matrix was already detached on the end of cut fibers.

**Figure 20:**
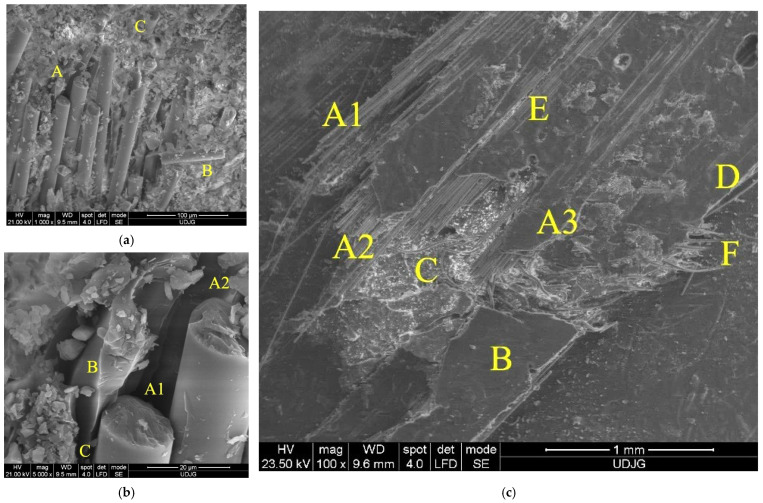
SEM images of the composite with 6 layers of quadriaxial fabric: (**a**,**b**)—panel face; (**c**)—panel back, after being hit by the impactor with diameter of 20 mm at an impact velocity v_3_ = 4 m/s.

Figure 20 presents the SEM images of the composite with six layers of quadriaxial glass fiber fabric after being hit by the impactor with a diameter of 20 mm at an impact velocity v_3_ = 4 m/s (the capital letters point out specific failure aspects of the composite):

(a) A—a detail from the panel face on the imprint contour, evidencing several fibers from the same yarn cut by shear loading and with their ends detached from the matrix; B—a fragment of glass fiber proving that the fibers could be broken in two points along their length, as reflected in Figure 9, by zero-stress segment for the main yarn in sublayer 7, at moment t = 8.75 × 10^−4^ s and also several moments later in the simulation for the other main yarns; C—a cut fiber that obviously has other orientations than those in point A;
(b) a detail of the same zone on the panel face: A1 and A2—the broken fibers by shear, B—the matrix that kept the shape of the broken and detached fiber and C—the crack that detached the matrix from a broken fiber;
(c) the back view of the panel, at smaller magnification (×100): A—partially broken yarns, A1—the fibers are broken at different lengths and the matrix is detached from these fibers, A2 and A3—the zones with broken fibers of the same yarn that kept the matrix attached to them, B—spalling of the matrix, C—the zone with a crushed matrix, D—visible local delamination, E—a small area with matrix exfoliations and F—cut fibers that were laterally underdriven by the local relative movement between the impactor and composite under load, as compared to the initial direction of the yarn; the last layer was locally damaged, but then, there was only a partial penetration.

**Figure 21:**
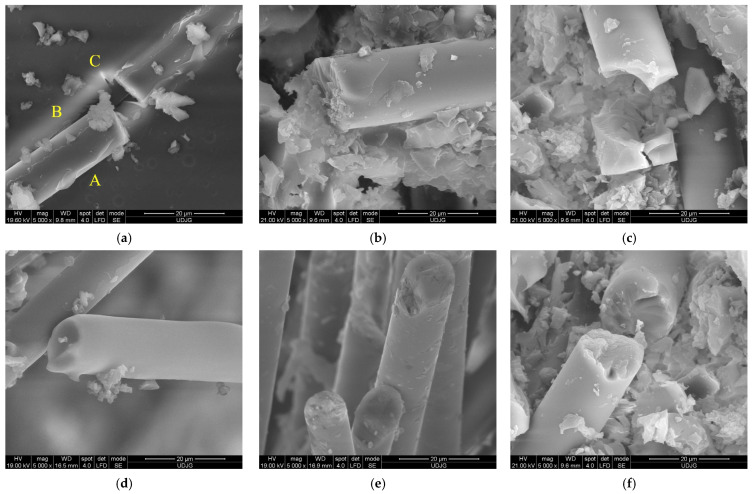
SEM images of the broken glass fibers. (**a**) 6-layer panel, impactor of 20 mm, v_1_ = 2 m/s, (**b**) 6-layer panel, impactor of 10 mm, v_3_ = 4 m/s, (**c**) 6-layer panel, impactor of 10 mm, v_3_ = 4 m/s, (**d**) 2-layer panel, impactor of 20 mm, v_3_ = 4 m/s, (**e**) 2-layer panel, impactor of 10 mm, v_3_ = 4 m/s and (**f**) 6-layer panel, impactor of 10 mm, v_3_ = 4 m/s.

Figure 21 presents, at the same magnification (×5000), the fiber breakage for different panels. Shear breakage seems to be similar for the fibers and does not give an indication of both test conditions and panel thickness. The failure mechanisms at the micro scale are as follows: (a) there is a broken fiber (A) still bonded in the matrix (B), but the fiber breakage also initiates cracks in the matrix (C); (b) there is a broken fiber with the cross-section almost perpendicular to the fiber length, meaning a dominant shear, as the fiber is still attached to the matrix on its end, but the matrix surrounding the fiber is cracked and fragmented, and on the fiber end, a superficial spalling is visible; (c) there is a fiber fragmented in more than one location, with the small fragment being cracked and the matrix being smashed and partially detached from this fiber: (d) and (e) there are fibers cut by shear, with cross-sections almost perpendicular to the fiber length, but one presents the trace of a detached chip; (f) there is a cut fiber, with one of the two fragments having changed its position, and the aspect of the breakage suggests a discontinuity in the fiber material (impurity or a high difference in composition).

## 6. Conclusions

This study presents a class of panels based on multi-axial glass fiber fabrics, designed and tested, which could offer protection in the range of 2–4 m/s, with impact energies up to 45 J. Tests with two hemispherical impactors, 10 mm and 20 mm in diameter, point out the influence of several factors: the thickness of the panels (by the number of quadriaxial glass fiber layers), the impact velocity and the impactor dimension.

At a velocity impact of 2 m/s (corresponding to an impact energy of 11 J), even the thinner panels (with two layers of quadriaxial glass fiber fabric, 1.64 mm thickness and a surface density of 3.51 kg/m^2^) have only partial penetration (damages on the panel face, without damage on panel back), but at an impact velocity of 4 m/s (corresponding to an impact energy of 45 J), only composite panels with six layers of quadriaxial fabric (5.25 mm thickness and a surface density of 9.89 kg/m^2^) present non-damaged back faces in the tests with both impactors. These results encourage the continuation of research on actual components for car and naval industries.

The analysis of the failure mechanisms of the panels was conducted using macrophotography and scanning electron microscope (SEM) images. These methods of investigation enabled the observation and in-depth understanding of how these composite materials behave under dynamic loading. An observation that is particularly noticeable is that the SEM images present failures which are characteristics to all performed tests, even though the differentiation may be conducted only for low magnification (×50, ×100), for the entire imprint or orifice, but failures of the fibers and matrix are visible with higher magnification (×500 to ×2000) and their aspects do not suggest a differentiation in impact parameters or composite thickness, aspects which were emphasized by macrophotography of high quality.

## Figures and Tables

**Table 1:** Elemental analysis of the glass fibers used in this study (average values) [20].

Wt%													
B	C	O	Na	Mg	Al	Si	S	Cl	K	Ca	Ti	Fe	Zn
Fiber cross-section													
29.39	24.75	9.03	0.38	0.42	3.55	14.21	1.75	0.20	0.22	8.88	0.50	2.19	4.43
Glass fiber jacket													
30.81	24.11	10.34	0.48	0.74	3.61	13.21	1.61	0.22	0.48	9.07	0.88	2.82	6.69

**Table 2:** Properties of the components of the resin Biresin^®^ CR82 and hardener CH80-2 [22].

Properties		Resin Component (A)	Hardener Component (B)
Components of the epoxy resin		Biresin^®^ CR82	Biresin^®^ CH80-2
Mixture ratio (parts)	Mass	100	27
Color		Transparent	In color till yellow or blue
Viscosity, 25 °C	mPa·s	~1600	
Density, 25 °C	g/mL	1.11	0.99
Mixture			
Pot life, 100 g/RT, approximative value		min	80
Viscosity of the mixture, 25 °C		mPa·s	600

**Table 3:** Mechanical and thermal properties of the epoxy resin [22].

Mechanical Properties of the Resin (after Ageing and Thermal Treatment)						
Resin Biresin^®^ CR82 (A)	With Hardener Biresin^®^ (B)		CH80-1	CH80-2	CH80-6	CH80-10
Tensile strength at breakage	ISO 527	MPa	94	90	84	82
Elastic modulus for traction	ISO 527	MPa	3000	3000	2900	2900
Strain at breakage	ISO 527	%	4.9	5.6	6.4	6.2
Flexural strength at breakage	ISO 178	MPa	140	130	127	118
Flexural modulus	ISO 178	MPa	3300	3200	2900	2800
Strength limit under compression	ISO 604	MPa	120	105	110	110
Density	ISO 1183	g/cm^3^	1.14	1.14	1.14	1.14
Shore hardness	ISO 868	-	D 85	D 85	D 85	D 85
Impact resistance	ISO 179	kJ/m^2^	38	66	55	56
Thermal properties of the resin (after ageing and thermal treatment)						
Temperature of deflection under load	ISO 75-1	°C	93	83	71	71
Glass transition temperature	ISO 11357	°C	97	90	83	85

**Table 4:** Characteristics of the produced panels.

No.	Fabric Mass	Panel Mass	Resin Mass *	Mass Ratio Fabric/Panel **	Surface Density ***	Thickness in 4 Points				
						1	2	3	4	Average
	[g]	[g]	[g]	-	[kg/m^2^]	[mm]				
Panel with 2 layers of quadriaxial fabric										
Panel 1/2	221.5	316	94.5	0.70	3.51	1.65	1.61	1.65	1.70	1.65
Panel 2/2	218.3	302.6	92.8	0.67	3.36	1.68	1.70	1.60	1.62	1.63
Average	219.9	309.3	93.65	0.68	3.43	1.66	1.65	1.62	1.66	1.64
Panel with 4 layers of quadriaxial fabric										
Panel 1/4	439.5	608.5	169	0.72	6.761	3.35	3.34	3.4	3.45	3.38
Placa 2/4	440	603.5	163.5	0.72	6.7	3.4	3.45	3.6	3.5	3.48
Average	439.75	606.0	166.25	0.72	6.73	3.37	3.39	3.50	3.47	3.43
Panel with 6 layers of quadriaxial fabric										
Panel 1/6	664	897	233	0.74	9.96	5.00	4.50	5.5	5.00	5.00
Panel 2/6	662	883.5	221.5	0.75	9.81	5.60	5.70	5.80	5.70	5.70
Average	663	890.25	227.25	0.74	9.89	5.3	5.1	5.65	5.35	5.35

* The resin mass = panel mass − fabric mass, meaning (column 2 − column 1); ** Mass ratio fabric/panel = Fabric mass/Panel mass, meaning (column 1/column 2); *** Surface density = Panel mass/Panel surface (0.09 m^2^).

**Table 5:** The average values of discussed parameters for impact with Φ10 mm impactor.

Panel	Thickness	F_max_	t_(Fmax)_	t_f_	t_(v=0)_	E_max_
	[mm]	[N]	[ms]	[ms]	[ms]	[J]
	v_1_ = 2 m/s					
2-layer	1.67	4075.0	2.34	9.37	4.66	11.09
4-layer	3.37	5832.1	2.53	5.67	2.82	11.01
6-layer	5.27	6396.7	2.00	4.51	2.21	10.89
	v_2_ = 3 m/s					
2-layer	1.66	3962.6	1.54	5.96	4.49	16.44
4-layer	3.73	8625.8	2.48	5.57	2.84	24.57
6-layer	5.27	9785.3	2.13	4.70	2.36	24.51
	v_3_ = 4 m/s					
2-layer	1.66	4673.6	1.35	- *	- *	14.3 *
4-layer	3.38	9529.4	1.75	4.92	2.52	43.54
6-layer	5.26	12,119.3	1.87	4.00	2.04	43.5

* Total penetration.

**Table 6:** The average values of discussed parameters for impact with impactor of Φ20 mm.

Panel	Thickness	F_max_	t_(Fmax)_	t_f_	t_(v=0)_	E_max_
	[mm]	[N]	[ms]	[ms]	[ms]	[J]
	v_1_ = 2 m/s					
2-layer	1.63	6143.6	3.22	6.80	3.56	11.42
6-layer	5.20	6618.6	1.89	4.66	2.20	11.40
	v_2_ = 3 m/s					
2-layer	1.70	9185.7	2.27	7.93	4.11	25.38
4-layer	3.37	11,711.4	2.37	4.84	2.40	25.44
6-layer	5.26	11,883.2	2.09	4.11	2.22	25.40
	v_3_ = 4 m/s					
2-layer	1.65	8868.5	1.46	- *	- *	44.7
4-layer	3.36	15,051.8	1.85	4.92	2.52	44.9
6-layer	5.27	18,250.2	1.74	4.00	2.04	45.1

* Total penetration.

**Table 7:** Types of failure of the tested panels with both impactors (Φ10 mm and Φ20 mm).

Number of Layers	v_1_ = 2 m/s	v_2_ = 3 m/s	v_3_ = 4 m/s
** Impactor Φ ** **10 mm**			
S2	PP	PP ⁕	PT
S4	PP	PP ⁕	PP ⁕
S6	PP	PP	PP
** Impactor Φ ** **20 mm**			
S2	PP	PP ⁕	PT
S4	PP	PP	PP ⁕
S6	PP	PP	PP

PT—total penetration, PP—partial penetration, PP ⁕—partial penetration with severe damages on the panel back, unrecommended for practical application.

## Data Availability

The data presented in this study are available on request from the corresponding author. All data from this research study are not publicly available due to their volume.
